# Microbubble-Assisted Ultrasound for Drug Delivery to the Retina in an Ex Vivo Eye Model

**DOI:** 10.3390/pharmaceutics15041220

**Published:** 2023-04-11

**Authors:** Charis Rousou, Nicky van Kronenburg, Andreas F. P. Sonnen, Marijke van Dijk, Chrit Moonen, Gert Storm, Enrico Mastrobattista, Roel Deckers

**Affiliations:** 1 Department of Pharmaceutics, Utrecht Institute for Pharmaceutical Sciences (UIPS), Utrecht University, Heidelberglaan 8, 3584 CS Utrecht, The Netherlands; 2 Imaging and Oncology Division, University Medical Center Utrecht, Heidelberglaan 100, 3584 CX Utrecht, The Netherlands; 3 Department of Translational Neuroscience, Brain Center, University Medical Center Utrecht, Heidelberglaan 100, 3584 CX Utrecht, The Netherlands; 4 Department of Pathology, Division of Laboratories, Pharmacy and Biomedical Genetics, University Medical Center Utrecht, Heidelberglaan 100, 3584 CX Utrecht, The Netherlands; 5 Department of Surgery, Yong Loo Lin School of Medicine, National University of Singapore, Singapore 119228, Singapore; 6 Department of Biomaterials Science and Technology, University of Twente, 7500 AE Enschede, The Netherlands

**Keywords:** ultrasound, microbubbles, intracellular accumulation, ex vivo perfused eye, retinal drug delivery

## Abstract

Drug delivery to the retina is one of the major challenges in ophthalmology due to the biological barriers that protect it from harmful substances in the body. Despite the advancement in ocular therapeutics, there are many unmet needs for the treatment of retinal diseases. Ultrasound combined with microbubbles (USMB) was proposed as a minimally invasive method for improving delivery of drugs in the retina from the blood circulation. This study aimed to investigate the applicability of USMB for the delivery of model drugs (molecular weight varying from 600 Da to 20 kDa) in the retina of ex vivo porcine eyes. A clinical ultrasound system, in combination with microbubbles approved for clinical ultrasound imaging, was used for the treatment. Intracellular accumulation of model drugs was observed in the cells lining blood vessels in the retina and choroid of eyes treated with USMB but not in eyes that received ultrasound only. Specifically, 25.6 ± 2.9% of cells had intracellular uptake at mechanical index (MI) 0.2 and 34.5 ± 6.0% at MI 0.4. Histological examination of retinal and choroid tissues revealed that at these USMB conditions, no irreversible alterations were induced at the USMB conditions used. These results indicate that USMB can be used as a minimally invasive targeted means to induce intracellular accumulation of drugs for the treatment of retinal diseases.

## 1. Introduction

Despite the advancements in ocular therapeutics, retinal drug delivery is yet one of the major challenges in ophthalmology [1,2,3]. Retinal diseases such as age-related macular degeneration and diabetic retinopathy are typically treated with intravitreal injection of drugs. This delivery method is unpleasant and is often accompanied by side effects, including retinal detachment, increase in intraocular pressure and retinal hemorrhages [4,5]. Another example of an open challenge is in the management of retinoblastoma, which is commonly treated with intravenous or intraarterial injection of chemotherapeutic agents. This therapeutic approach is often associated with the inadequate delivery of drugs to the target cells, resulting in the presence of subretinal and vitreous seeds that cause recurrent and chemoresistant tumors [6]. These examples highlight the need for novel and less invasive drug delivery approaches that enable targeted treatment of retinal diseases. To overcome the current challenges in retinal drug delivery, several approaches were developed, such as the use of nanoparticles and sustained drug release systems [7,8,9,10].

Another approach studied by numerous scientific groups for drug delivery in the eye and other organs is the combination of ultrasound with microbubbles (USMB) [11,12,13]. Microbubbles are gas-filled microspheres 0.5–10 μm in diameter, widely used as ultrasound contrast agents [14,15]. When microbubbles are exposed to ultrasound waves, they induce a number of effects to the cells in vicinity. Among the effects caused are sonopermeation (i.e., the formation of pores in cell membranes/sonoporation and enhanced endocytosis) and rearrangement of intercellular junctions in endothelial or epithelial barriers [16,17]. Exploiting these effects, several studies demonstrated the potential of USMB to enhance the intracellular uptake of therapeutics (e.g., small molecule drugs, genes, antibodies, nanoparticles) and their permeability across biological barriers [13,18,19,20,21,22].

Among other applications in ophthalmology, USMB was shown to improve the delivery of drugs to the retina by transiently permeabilizing the blood–retina barrier (BRB). The in vivo animal studies of Hirokawa et al. and Park et al. focused on the USMB-induced extravasation of small molecules (fluorescein; molecular weight 332 Da, and gadolinium; molecular weight 938 Da) from the retinal blood vessels [23,24]. Park et al. reported that BRB permeabilization was restored within 3.5 h after USMB treatment, highlighting the reversibility of the effect. Furthermore, USMB-induced extravasation of macromolecules (molecular weight 66–970 kDa) and delivery of a recombinant adeno-associated virus to retina Müller glial cells were recently also shown [25]. While the above studies provided invaluable insights on the activity of USMB on the permeabilization of the BRB, the effect on intracellular drug delivery is yet to be investigated. 

This ex vivo study aimed to examine the potential and safety of USMB to induce intracellular accumulation of membrane-impermeable fluorescent molecules, both small (SYTOX^TM^ green) and large (4 and 20 kDa dextrans) in the posterior segment of the eye. To this end, a clinical ultrasound system (iU22) was used in combination with clinically approved microbubbles (SonoVue^TM^) to perform USMB treatment in arterially perfused porcine eyes.

## 2. Materials and Methods

### 2.1. Chemicals

Fluorescent-conjugated dextrans (TRITC-dextran 4400 Da; T1037 and TRITC-dextran 20,000 Da; 73766), histology mounting medium containing DAPI (Fluoroshield^TM^ with DAPI, F6057), paraformaldehyde (PFA, 158127), 10% buffered formalin solution (MFCD00003274) and sucrose (S8501) were acquired from Sigma-Aldrich (Steinheim, Germany). SYTOX^TM^ green (S7020) was obtained by ThermoFisher Scientific (Waltham, MA, USA).

### 2.2. Preparation and Cannulation of Perfused Eyes

Enucleation and cannulation of porcine eyes was performed as previously described [26]. In brief, eyes were dissected directly after animal termination and transported to the laboratory within one hour in heparinized (5 IU/mL) saline (0.9% NaCl in water) at 0 °C. The ophthalmic artery was localized and cannulated using a 0.61 mm (outer diameter) tube. Subsequently, the ophthalmic artery was perfused with saline using a syringe pump (pump model 33, Harvard Apparatus, Cambridge, MA, USA) at a flow rate of 0.1 mL/minute. Elimination of blood from the retinal blood vessels during perfusion indicated successful cannulation of the ophthalmic artery. Eyes with a successfully cannulated ophthalmic artery were further used for treatment with USMB.

### 2.3. Perfusion of Microbubbles and Treatment with USMB

SonoVue^TM^ (Bracco International B.V., Amsterdam, The Netherlands) was prepared according to the manufacturer’s instructions, producing sulfur hexafluoride-filled phospholipid-coated microbubbles with a mean bubble diameter of 2.5 μm and a concentration of 1–5 × 10^8^ microbubbles/mL [27]. Microbubbles were kept at 4 °C in-between use, resuspended before every use and used within 4 h after preparation.

**Figure 1:**
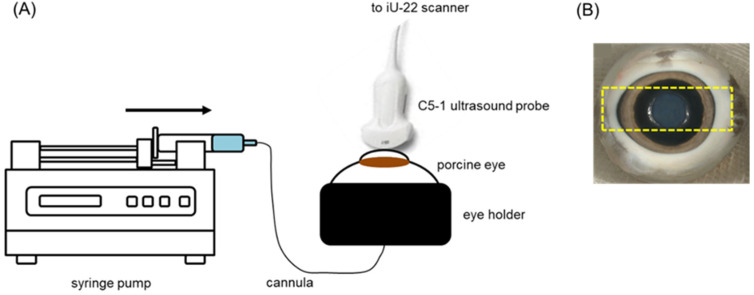
(**A**) Experimental setup used in USMB experiments. (**B**) Image of a porcine eye. Yellow rectangle indicates the orientation of the C5-1 probe during ultrasound imaging and USMB treatment.

**Figure 2:**
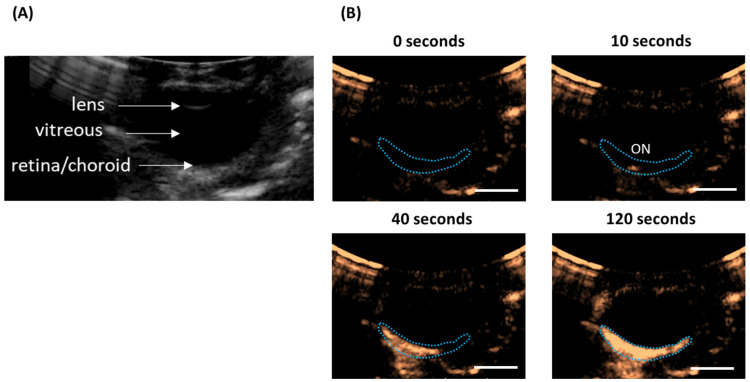
(**A**) Ocular structures of a porcine eye as imaged with ultrasound in B-mode. (**B**) Time-lapse ultrasound contrast images of porcine eye during injection of microbubbles. Ten seconds after injection, microbubbles enter the posterior segment of the eye in the area of the optic nerve head. Two minutes after injection, microbubbles circulate in the retina and choroid. Blue dotted line indicates the retina and choroid, scale bar: 1 cm.

**Figure 3:**
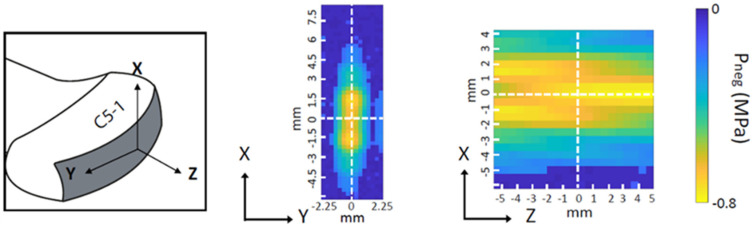
Pressure field maps of the C5-1 probe measured in PW Doppler mode. Middle: transversal plane, right: axial plane. Adapted from [28].

Ultrasound imaging and USMB treatment were performed using a clinical system (iU22, Philips Medical Systems Nederland B.V., Best, The Netherlands) and the C5-1 probe. Using an articulated arm (MA60103, Noga Engineering & Technology, Shlomi, Israel), the ultrasound probe was fixed on the cornea (Figure 1A,B) with a small amount of ultrasound gel in-between the cornea and the probe to ensure acoustic coupling. Prior to USMB treatment, ocular structures were visualized with B-mode imaging (Figure 2A). Subsequently, eyes were perfused with a solution containing microbubbles (diluted 10-times by volume using PBS), SYTOX^TM^ green (final concentration 2 μM in PBS) and TRITC-dextran (final concentration 0.2 mM in PBS). During perfusion, circulation of microbubbles in the posterior segment of the eye was confirmed by imaging the eye in contrast mode at mechanical index (MI) of 0.05 (Figure 2B). Subsequently, USMB therapy was performed in Pulsed Wave (PW) Doppler mode (transmission frequency: 2.25 MHz, sample volume: 10 mm). Circulation of microbubbles, SYTOX^TM^ green and TRITC-dextran was continuous during USMB treatment. During USMB treatment, the PW Doppler cursor was aligned at the center of the retina and the optic nerve head was used as spatial reference. The acoustic field of the C5-1 ultrasound probe in PW Doppler mode was measured using a 0.2 mm needle hydrophone (Precision acoustics Ltd., Dorset, UK) in degassed water [28] (Figure 3). During measurement of the acoustic field, the hydrophone was positioned 3 cm away from the surface of the ultrasound probe. Measurements were acquired at MI 0.8.

USMB treatment was performed for 2 min at two different MIs (MI 0.2 and 0.4 corresponding to peak-negative pressures of 0.3 and 0.6 MPa, respectively). Contrast images were acquired before and after treatment. Eyes perfused with the same solution but without microbubbles were treated at the same conditions and were used as control samples. Signal intensity from microbubbles within the PW Doppler cursor was determined using contrast images of eyes treated at MI 0.2 and 0.4, before and after USMB treatment. A region of interest (ROI) was drawn in the area within the PW Doppler cursor and signal intensity within the ROI was automatically measured (ImageJ software, National Institutes of Health, Bethesda, MD, USA). Immediately after USMB treatment, eyes were perfused for 30 min with a solution containing SYTOX^TM^ green and TRITC-dextran at the same concentrations as above and were subsequently fixed with perfusion of PFA for 1 min. At the end of the experiment, periocular tissues were removed, eyeballs were immersed in PFA and preserved at 4 °C for 48 h until the preparation of flat mounts.

### 2.4. Preparation of Flat Mounts and Cryosectioning

Flat mounts were prepared using fixed eyes. An incision was made in the anterior eye 2–3 mm away from the limbus and anterior ocular structures (cornea, iris, lens) were removed. Four incisions were made radially to the optic nerve head and the vitreous body was removed. Subsequently, flat mounts were illuminated with green fluorescent light (excitation 510–540 nm, NightSea, Lexington, MA, USA) and macroscopic images were acquired through the viewing shield made of a 600 nm longpass filter material. Samples were then preserved in 30% sucrose in water solution (*w*/*v*) at 4 °C overnight. Subsequently, flat mounts were immersed in embedding compound for cryosectioning (OCT) and were preserved at −80 °C until sectioning. Samples were serially sectioned at 10 μm in the sagittal plane (parallel to the direction of ultrasound beam propagation) using a cryostat (CM1950, Leica, Amsterdam, The Netherlands). Sections were sealed with a coverslip using mounting medium containing DAPI.

### 2.5. Fluorescence Microscopy and Quantification of the Number of Cells with Intracellular Accumulation

Tissue sections were imaged using a fluorescence confocal microscope (Leica TCS SP8X, Leica, Amsterdam, The Netherlands) in four channels (excitation 360 nm; emission 410–480 nm for DAPI, excitation 504 nm; emission 515–546 nm for SYTOX^TM^ green, excitation 550 nm; emission 565–650 nm for TRITC-dextrans, and phase contrast, 63× objective, image format 2048 × 2048 pixels; speed: 100; line average: 4).

Quantification of the number of cells with intracellular accumulation as a result of USMB treatment was performed with the ImageJ software. A ROI was drawn in blue channel (DAPI) images surrounding the cells that form the blood vessel lumens. Within this ROI the total number of cells in the blue channel and the number of SYTOX^TM^ green-positive cells in the green channel were automatically determined. Subsequently, the percentage of SYTOX^TM^ green-positive cells over the total number of cells within the ROI was calculated.

## 3. Histology

For the needs of histological analysis, heparinized porcine blood (1 IU/mL) was used as perfusate. After treatment with USMB, one eye from each experimental group (MI 0.2 and 0.4) was perfused with blood for 30 min and was fixed with PFA as described above. Additionally, one eye that was exposed to USMB at MI 0.8 was used as a positive control, as extravasation of erythrocytes was previously reported at this acoustic pressure [24]. One eye that was perfused with blood for 30 min but was not exposed to USMB was used as a negative control.

Subsequently, the vitreous body was removed using a syringe, whilst the ocular cavity was infused with formalin. Eyes were then cleaved along the optic nerve—pupil axis, were fixated in formalin and were embedded in paraffin. Four micrometer thick sections were prepared for each half eye. Several hematoxylin and eosin (H&E) stained sections acquired from different depths were prepared for examination. Slides were then digitized using a digital slide scanner (NanoZoomer S360, Hamamatsu City, Japan). The authors (AS and MvD) who evaluated the histology were blinded to the USMB exposure parameters.

## 4. Statistical Analysis

Statistical analysis was performed using the GraphPad Prism software (version 8.0.1, GraphPad, San Diego, CA, USA), assuming that the samples follow non-parametric distribution. Statistically significant differences in the percentage of cells with intracellular accumulation were calculated between USMB-treated and samples treated only with ultrasound using Mann–Whitney test. Data in the graphs are shown as mean ± SEM. Statistically significant differences between groups are annotated with asterisks by using * for *p* < 0.05 and ** for *p* < 0.005.

## 5. Results

### 5.1. Microbubble Circulation in the Posterior Segment of the Eye

**Figure 4:**
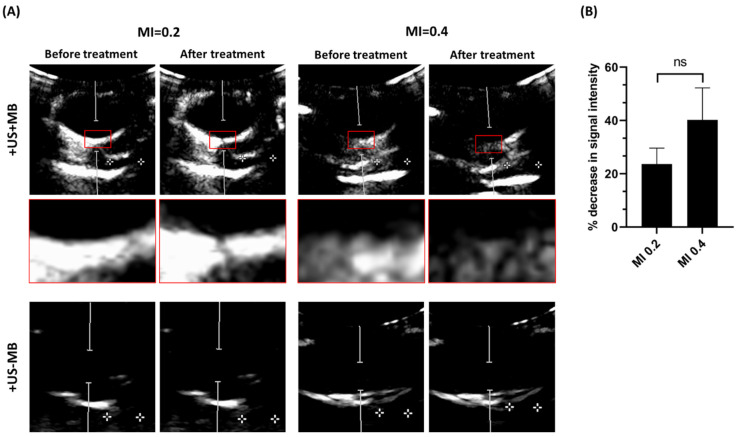
(**A**) Ultrasound contrast images from porcine eyes treated with UMSB (+US+MB) or ultrasound only (+US−MB) at MI 0.2 and 0.4. Red boxes indicate enlargement of the focal area of the probe. Contrast signal was minimally (MI 0.2) and strongly (MI 0.4) decreased after USMB treatment. Distance between the two crosses is 1 cm. (**B**) Percentage of decrease in signal intensity of microbubbles within the PW Doppler cursor area after treatment with USMB at MI 0.2 and 0.4 (n = 4), ns: not significant.

Microbubble circulation in the porcine retina and choroid was imaged with ultrasound in contrast mode before and after USMB treatment. Figure 4A demonstrates the difference in intensity of microbubble signal for eyes treated with USMB (+US+MB) at MI 0.2 and 0.4 before and after treatment (red boxes). At MI 0.2, the difference between before and after USMB treatment was minimal. On the other hand, at MI 0.4, the microbubble signal was strongly decreased in intensity after USMB treatment at a large area (the weak microbubble signal on the left side of the images from the eye treated with USMB at MI 0.4 was likely due to an occluded blood vessel). This area was aligned with the PW Doppler cursor (white cursor), which corresponded to the focus of the probe. This observation is in line with microbubble behavior earlier reported in literature: at MI 0.2, microbubbles more likely undergo stable cavitation, whilst at MI 0.4, the higher acoustic pressure also induces microbubble collapse (inertial cavitation) [29]. However, due to microbubble polydispersity, both stable and inertial cavitation likely occur simultaneously even at higher MIs.

Signal intensity from microbubbles within the PW Doppler cursor was quantified using contrast images of eyes treated at MI 0.2 and 0.4, before and after USMB treatment. At MI 0.2, microbubble intensity was decreased by 23.7 ± 5.2%. The corresponding value for MI 0.4 was 40.2 ± 9.8%. However, the difference between the two groups was not statistically significant.

### 5.2. Intracellular Accumulation of Model Drugs in the Retina and Choroid

**Figure 5:**
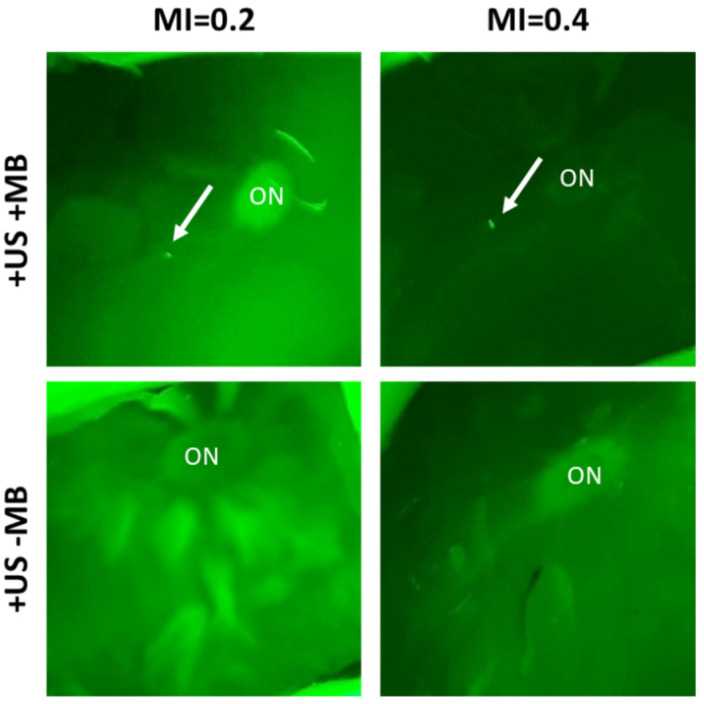
Macroscopic images of retina flat mounts of eyes during excitation with a green fluorescent light. Top: eyes treated with USMB (left: MI 0.2, right: MI 0.4), bottom: eyes exposed to ultrasound but no microbubbles. White arrows indicate the spots where SYTOX^TM^ green-positive cells were present. Retinal detachment is an artefact of flat mount preparation. ON: optic nerve head.

Intracellular accumulation of model drugs (SYTOX^TM^ green; molecular weight 600 Da, TRITC dextrans; molecular weight 4 and 20 kDa) as a result of USMB treatment was investigated in ex vivo porcine eyes. SYTOX^TM^ green is a hydrophilic, membrane impermeable fluorescent dye with small molecular weight (600 Da), commonly used as a model drug in studies investigating the effect of USMB on intracellular drug accumulation [28,30,31]. After USMB treatment, the eyes were perfused with a solution containing either SYTOX^TM^ green and 4 kDa dextran or SYTOX^TM^ green and 20 kDa dextran. Subsequently, the eyes were fixed and flat mounts were prepared. During preparation of flat mounts, the retina was macroscopically visualized using green fluorescence light (Figure 5). In three out of the five eyes that were treated with USMB a single, a green spot was observed at the center of the retina in close proximity to the optic nerve head (Figure 5, upper row). From those three eyes where a green spot was observed, one was perfused with SYTOX^TM^ green/4 kDa TRITC-dextran and two eyes were perfused with SYTOX^TM^ green/20 kDa TRITC-dextran. The location of these spots suggests, as they were spatially overlapping with the focus of the ultrasound beam, that they likely consisted of a group of cells with intracellular uptake of SYTOX^TM^ green. To further examine these areas, the tissue including this spot and the optic nerve was isolated, sectioned and imaged with a fluorescent microscope. These spots were not observed in any of the eyes that were exposed to ultrasound without the presence of microbubbles (Figure 5, bottom row).

**Figure 6:**
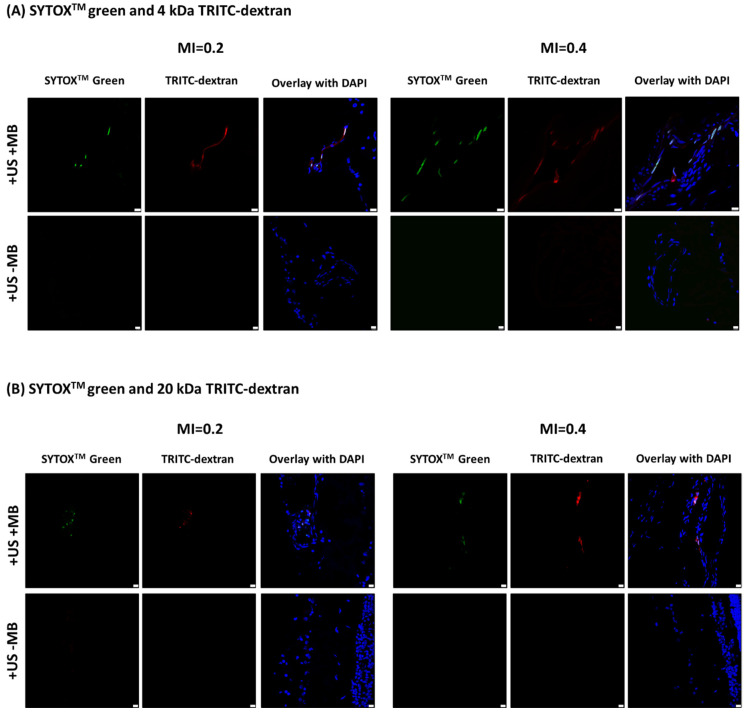
Fluorescence microscopy images showing intracellular accumulation of (**A**) SYTOX^TM^ green and 4 kDa TRITC-dextran and (**B**) SYTOX^TM^ green and 20 kDa TRITC-dextran after exposure to USMB. “TRITC” indicates a fluorophore with red color. Blue in overlay images: DAPI, scale bar: 10 μm.

Fluorescence microscopy of tissue sections revealed that USMB induced intracellular uptake of SYTOX^TM^ green and fluorescent dextrans (Figure 6). Fluorescence of SYTOX^TM^ green and dextrans was observed microscopically (i.e., with the fluorescence microscope) even in the two out of five eyes that were treated with USMB but showed no green fluorescent signal macroscopically (i.e., visible for the inspector’s eyes). In the USMB-treated eyes where a green fluorescent spot was visible macroscopically (three out of five eyes), intracellular accumulation of SYTOX^TM^ green was also seen microscopically in the area of the green spot, with no signs of autofluorescence. Microscopic observation of the accumulation of fluorescent molecules was not observed in any of the eyes that were treated with ultrasound without microbubbles. 

**Figure 7:**
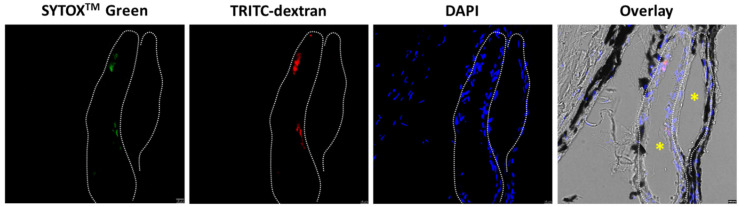
Example of induced intracellular accumulation in cells lining blood vessels in the choroid after treatment with USMB at MI 0.4. Yellow asterisks in the overlay image indicate the lumen of choroidal blood vessels. White dotted line indicates the area of cells lining the blood vessel lumen. Overlay image is a combination of fluorescent and phase contrast images. Green is indicated by the “SYTOX Green name”. TRITC and DAPI are widely used fluorophores with red and blue colors, respectively. Scale bar: 10 μm.

Intracellular accumulation in USMB-treated eyes was observed in cells lining the lumen of blood vessels in the retina and choroid (Figure 6 and Figure 7). Considering that microbubbles were injected into the ophthalmic artery, the cells lining the blood vessel walls were in contact with microbubbles during treatment and, therefore, were directly exposed to microbubble oscillations. In all cells with intracellular accumulation, both SYTOX^TM^ green and one of the two dextrans were present. SYTOX^TM^ green was localized only in the cell nucleus whilst dextrans were present both inside the cytosol and the nucleus. No evidence of extravasation of dextrans in the retina or choroid was found.

**Figure 8:**
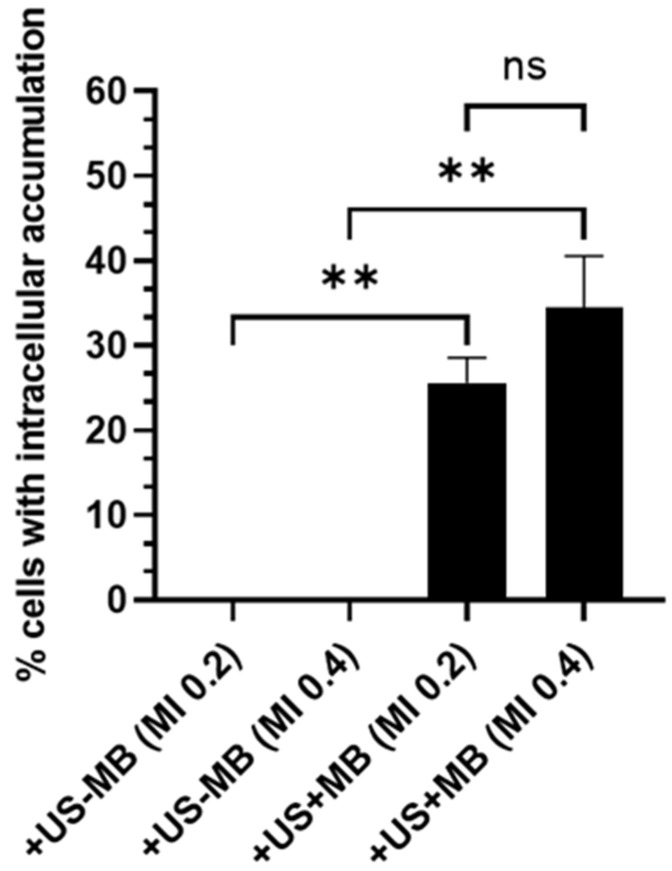
Quantification of the percentage of cells with intracellular accumulation of SYTOX^TM^ green over the total number of cells lining blood vessels (n = 5), ** for *p* < 0.005, ns: not significant.

A quantification of the number of cells with intracellular accumulation as a result of USMB treatment was performed by calculating the number of SYTOX^TM^ green-positive cells over the total number of cells lining the blood vessel, which revealed that USMB induced a significant increase in the intracellular accumulation of this model drug (Figure 8). Specifically, eyes treated at MI 0.2 had, on average, 25.6 ± 2.9% of SYTOX^TM^ green-positive cells, and eyes treated at MI 0.4 had 34.5 ± 6.0% positive cells. The difference between MI 0.2 and 0.4 was, however, not significant. Since no accumulation was observed in the eyes treated with ultrasound only, for the needs of the statistical analysis, the number of SYTOX^TM^ green-positive cells for the corresponding groups (i.e., +US−MB) was set to zero. The number of SYTOX^TM^ green-positive cells in the eyes treated with ultrasound without microbubbles (+US−MB) in Figure 6 and Figure 7 was zero.

## 6. Histology Analysis

**Figure 9:**
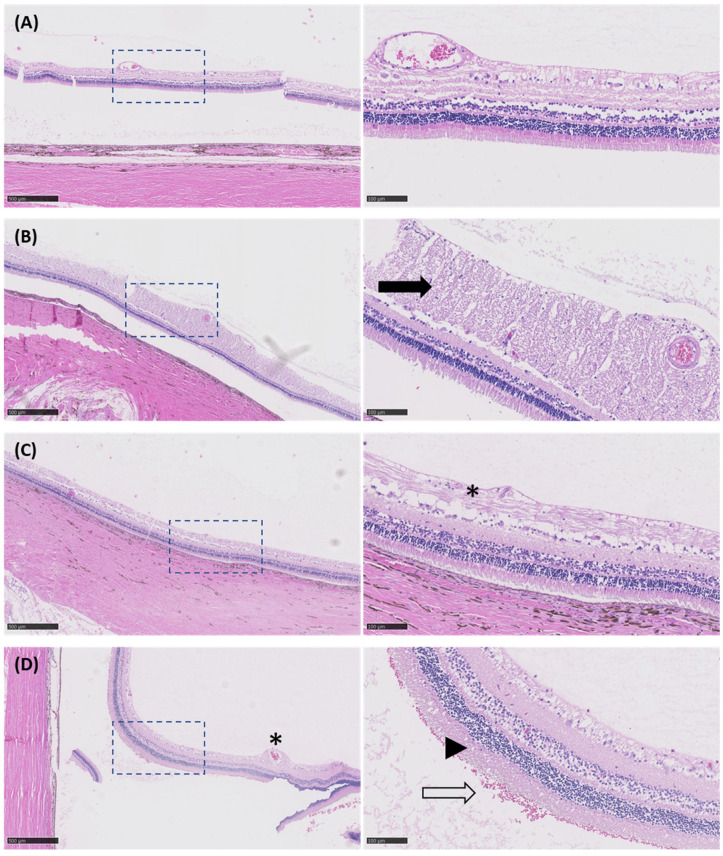
Microphotographs of H&E-stained sections from eyes exposed to different USMB conditions. Images on the right (20× magnification, scale bar: 100 μm) are high magnification views of dashed-line boxes shown on the left (5× magnification, scale bar: 500 μm). (**A**) Untreated eye (i.e., no USMB) with no signs of retinal damage. (**B**) Eye treated with USMB at MI 0.2 showing some edematous changes (black arrow) in the proximity of the optic nerve. (**C**) In the eye treated with USMB at MI 0.4, focal degenerative vacuolization with fibrinous exudate was observed (asterisk). (**D**) The most pronounced damage was seen in the eye treated with USMB at MI 0.8. Focal degenerative vacuolization with fibrinous exudate was seen around the retinal blood vessel (asterisk), erythrocytes were present in the subretinal space (hollow arrow) and loss of cell boundaries was seen in the photoreceptor layer (arrowhead).

A histological examination was performed on three eyes that were treated with USMB (MI 0.2, 0.4 and 0.8) and one untreated eye that served as negative control (Figure 9). In the eye treated with USMB at MI 0.2, limited damage was observed, with edematous changes in the proximity of the optic nerve being the most pronounced (Figure 9B). In the eye treated at MI 0.4, focal degenerative vacuolization with fibrinous exudate on the foreground was seen (Figure 9C). At the highest USMB conditions (MI 0.8), there was focal degenerate vacuolization with fibrinous exudate around retinal blood vessel (Figure 9D). Further, at MI 0.8, focal vacuolization of retinal cells and focal subretinal accumulation of erythrocytes was seen, and the cellular boundaries of photoreceptors was lost, indicating cellular damage. All eyes suffered from preparation artifacts, such as detachment of the neural retina from the retinal pigment epithelium and choroid. No focal transretinal bleeding was observed.

## 7. Discussion

Targeting the retina from blood circulation is a less invasive alternative to intravitreal injections, the latter being the most frequently encountered drug delivery method in the treatment of retinal degenerative diseases. USMB was previously proven as a method to transiently permeabilize the BRB and to allow the extravasation of (model) drugs from the blood circulation [23,24,25]. In this study, the intracellular accumulation of model drugs as a result of USMB treatment was investigated using an ex vivo porcine eye model. Fluorescent molecules with molecular weight varying between 600 Da and 20 kDa were chosen to be used as model drugs, as they cover the molecular weights of a large range of clinically employed drugs. To evaluate the applicability of USMB, a clinically approved ultrasound system and clinically used microbubbles for ultrasound imaging was utilized. Intracellular accumulation of SYTOX^TM^ green (molecular weight 600 Da) and fluorescent dextrans (molecular weight 4 kDa and 20 kDa) were simultaneously observed in eyes exposed to USMB at MI 0.2 and 0.4, but not in eyes treated with ultrasound only. 

The number of SYTOX^TM^ green-positive cells were significantly increased in eyes treated with USMB at MI 0.2 and 0.4 compared to eyes that received ultrasound but no microbubbles. In contrast to previous studies [23,24,25], no evidence of extravasation of model drugs from the blood vessels into the retina was seen as a result of BRB permeabilization. This difference might be attributed to the lower MIs used in our study. These MIs were chosen because they are lower than the MIs earlier reported to cause retinal damage after USMB treatment [23,24,25]. Similar to our observation, Hirokawa et al. also did not observe any extravasation of fluorescein from retinal blood vessels after treatment of in vivo rabbit eyes with USMB (MI 0.2, frequency 2 MHz, Definity^®^ microbubbles), though some alterations in the diameter of uveal blood vessels were detected [23]. However, in their study, uveal endothelial cells were not microscopically examined and, therefore, no conclusion can be made concerning the intracellular accumulation of fluorescein at an MI 0.2. On the other hand, Park et al. reported the presence of gadolinium in the vitreous as a result of extravasation from the retina 10 min after USMB treatment [24]. However, the ultrasound pressures used in their study were much higher than ours (MI 0.98 to 1.32, frequency 0.7 MHz, Definity^®^ microbubbles). Similarly, Touahri et al. observed extravasation of Evans blue in the retina 30 min after USMB treatment using MIs between 0.55 and 0.87 (frequency 1 MHz, Definity^®^ microbubbles) [25].

The accumulation of model drugs was seen in cells lining blood vessels in the retina (Figure 6A,B; MI 0.2) but also in the choroid (Figure 6B; MI 0.4 and Figure 7). Since microbubbles were circulating in the blood vessels, these cells were presumably endothelial cells [32]. However, additional experiments using endothelial cell-specific markers (such as CD31) are needed in order to determine with certainty the exact cell type. Intracellular delivery of drugs in the endothelium of the retinal and choroidal blood vessels using USMB could be beneficial for the treatment of certain retinal pathologies. An example is retinal neovascularization as a result of diabetic retinopathy. Retinal neovascularization is a condition where abnormal, leaky blood vessels appear on the surface of the retina leading to vitreous hemorrhage and retinal detachment. Endostatin, a C-terminal fragment of collagen XVIII with molecular weight of 20 kDa, could be used in combination with USMB to inhibit endothelial cell proliferation and angiogenesis [33,34]. Another example of the use of USMB is to enhance the intracellular delivery of chemotherapeutic drugs, such as vincristine (molecular weight 825 Da), etoposide (molecular weight 589 Da) and carboplatin (molecular weight 370 Da), into retinal tumor cells for the treatment of retinoblastoma in children [35].

In our study, USMB treatment was performed using a clinical ultrasound system in PW Doppler mode. PW Doppler was previously utilized in therapeutic USMB to induce intracellular accumulation of chemotherapeutic agents and blood–brain barrier permeabilization [28,36,37]. To the best of our knowledge, this is the first time that PW Doppler was used to facilitate retinal drug delivery. The advantage of this method is that clinical translation can be easier facilitated compared to previous studies that used ultrasound systems which are only used for preclinical research. [24,25]. Furthermore, performing USMB treatment using an ultrasound scanner provides the advantage of simultaneous imaging of the ocular structures (in B-mode) and microbubbles (in contrast mode). In a previous study, a clinical ultrasound system (B-mode) was used for USMB treatment [23]. However, the PW Doppler mode used in our study enables to limit microbubble cavitation by focusing the ultrasound beam in an area of a few millimeters (Figure 3), and therefore, has more control of the size of the treated area. In addition, a larger number of cycles per pulse is more beneficial for therapeutic USMB [28,38], making PW Doppler settings (32 cycles per pulse [28]) more suitable for drug delivery applications over B-mode (typically 4–5 cycles per pulse [39]). 

Achieving drug delivery in the retina, whilst adverse effects are avoided, is of utmost importance. In our study, USMB treatment at low MIs (0.2 and 0.4) induced only mild alterations in the retina (edema, fibrinous exudate and vacuolization), which are reversible. On the other hand, at MI 0.8, more severe side-effects were observed (subretinal erythrocytes, loss of photoreceptor boundaries) in addition to degenerate vacuolization, with loss of cell boundaries being the most severe, as it indicates photoreceptor cell death. USMB treatment at MI 0.8 was used as a positive control, as it was previously reported that treatment at that this MI induces irreversible retinal damage [24].

One limitation of our study was that the pressure field map of the ultrasound probe was measured in free field (i.e., inside a water tank); therefore, the interaction of ultrasound waves (e.g., attenuation, refraction) with the anterior ocular tissues, such as the cornea and lens, and vitreous was not taken into account [40,41,42]. Measurement of the acoustic field in PW Doppler mode in the presence of the anterior eye would allow for a more accurate determination of the acoustic energy in the posterior eye during USMB treatment. Furthermore, it is possible to estimate the in situ values of intensity or acoustic pressure using knowledge of the propagation path and tissue-specific derating factors [41,43]. Another limitation of our study was that microbubble backscattered signals were not monitored. In the future, an integrated system for detection of microbubble cavitation regime could be used during treatment in order to provide more insights on microbubble activity and the safety of USMB in retinal drug delivery. An example of such a system is the clinical ultrasound device previously used to induce controlled permeabilization of the blood–brain barrier to enhance the delivery of carboplatin in a rat glioma model [44]. Another interesting research question that arises from this study is on the effects induced by USMB on the posterior segment of the eye, whether permeabilization of the BRB and extravasation of model drugs occurs along with intracellular accumulation, and whether the extent of these effects is driven by the ultrasound pressure applied.

## 8. Conclusions

In conclusion, this study showed that USMB can be used to induce intracellular accumulation of model drugs in cells lining the blood vessels in the retina and choroid. The USMB treatment conditions used in this study improved the intracellular accumulation of model drugs while causing only mild and reversible histological alterations in the retina. This technique could provide a minimally invasive method to deliver drugs to the posterior segment of the eye from the blood circulation for the treatment of retinopathies, as an alternative to intravitreal injections.

## Data Availability

The data presented in this study are available on request from the corresponding author.
